# Apoptosis Signal-Regulating Kinase 1 Is Involved in Brain-Derived Neurotrophic Factor (BDNF)-Enhanced Cell Motility and Matrix Metalloproteinase 1 Expression in Human Chondrosarcoma Cells

**DOI:** 10.3390/ijms140815459

**Published:** 2013-07-25

**Authors:** Chih-Yang Lin, Sunny Li-Yun Chang, Yi-Chin Fong, Chin-Jung Hsu, Chih-Hsin Tang

**Affiliations:** 1 Graduate Institute of Basic Medical Science, China Medical University, Taichung 404, Taiwan; E-Mails: p123400@hotmail.com (C.-Y.L.); liyunchang@mail.cmu.edu.tw (S.L.-Y.C.); 2 School of Chinese Medicine, China Medical University, Taichung 404, Taiwan; E-Mails: yichin.fong@msa.hinet.net (Y.-C.F.); jeffrey5991@gmail.com (C.-J.H.); 3 Department of Orthopaedics, China Medical University Hospital, Taichung 404, Taiwan; 4 Department of Pharmacology, School of Medicine, China Medical University, Taichung 404, Taiwan; 5 Department of Biotechnology, College of Health Science, Asia University, Taichung 404, Taiwan

**Keywords:** BDNF, TrkB, ASK1, MMP-1, chondrosarcoma

## Abstract

Chondrosarcoma is the primary malignancy of bone that is characterized by a potent capacity to invade locally and cause distant metastasis, and is therefore associated with poor prognoses. Chondrosarcoma further shows a predilection for metastasis to the lungs. The brain-derived neurotrophic factor (BDNF) is a small molecule in the neurotrophin family of growth factors that is associated with the disease status and outcome of cancers. However, the effect of BDNF on cell motility in human chondrosarcoma cells is mostly unknown. Here, we found that human chondrosarcoma cell lines had significantly higher cell motility and BDNF expression compared to normal chondrocytes. We also found that BDNF increased cell motility and expression of matrix metalloproteinase-1 (MMP-1) in human chondrosarcoma cells. BDNF-mediated cell motility and MMP-1 up-regulation were attenuated by Trk inhibitor (K252a), ASK1 inhibitor (thioredoxin), JNK inhibitor (SP600125), and p38 inhibitor (SB203580). Furthermore, BDNF also promoted Sp1 activation. Our results indicate that BDNF enhances the migration and invasion activity of chondrosarcoma cells by increasing MMP-1 expression through a signal transduction pathway that involves the TrkB receptor, ASK1, JNK/p38, and Sp1. BDNF thus represents a promising new target for treating chondrosarcoma metastasis.

## 1. Introduction

The brain-derived neurotrophic factor (BDNF) is a physiologically important nerve growth factor that is a member of the neurotrophin family of growth factors [1]. BDNF binds and subsequently activates a tyrosine kinase receptor, tropomyosin related kinase B (TrkB), which is important for the development of the nervous system. Recent studies have suggested a major role for BDNF in cancer cell proliferation, survival, differentiation, and invasiveness [2,3]. Furthermore, BDNF was shown to be expressed in several tumor types such as breast cancer, lung cancer, colon cancer, hepatocellular carcinoma, and head and neck squamous cell carcinoma [4–10]. Therefore, BDNF may be a novel target for tumor detection and chemotherapy.

Chondrosarcomas are malignant tumors of cartilage tissue that do not respond well to chemotherapy or radiotherapy treatment [11]. They are the second most frequent primary malignant type of bone tumors, can occur at any point between 10 and 80 years of age with approximately two-thirds of chondrosarcoma patients being male [12], and usually appear on the scapula, sternum, ribs, and pelvis [13]. In general, this mesenchymal malignancy has a poor prognosis. Clinically, surgical resection remains the primary mode of therapy for chondrosarcoma. Due to the lack of an effective adjuvant therapy, the identification of novel molecular mechanisms is important for developing adequate treatment strategies for this disease.

The metastasis of tumor cells is a complex and multistage process. Experiments have confirmed that cell metastasis is facilitated through changes in cell-cell adhesion properties, rearrangement of the extracellular matrix (ECM) environment, suppression of anoikis, and reorganization of the cytoskeleton [14]. Matrix metalloproteinases (MMPs) play important roles in these processes, as they activate endopeptidases that are capable of enzymatically degrading the ECM proteins comprising the tumor microenvironment [15]. Twenty-three members of the human MMP gene family have been identified to date. Studies have shown that MMP-1, MMP-2, MMP-3, MMP-9, and MMP-13 exhibit high degrees of expression in human chondrosarcoma cells [16]. Moreover, MMP-1 has been found to play a role in the ECM degradation associated with cancer metastasis, and is therefore a prognostic factor for human chondrosarcoma [17].

Apoptosis signal-regulating kinase 1 (ASK1) is a member of the mitogen-activated protein kinase (MAPK) kinase kinase (MKKK) family that functions in activating the c-jun *N*-terminal kinase (JNK) and p38 signaling pathways. ASK1 affects multiple cellular functions [18] including survival, differentiation, and the innate immune response [19–21], and has been reported to regulate vascular smooth muscle cell migration [22]. In addition, ASK1 plays a crucial role in regulating MMP-1 expression [23]. However, the role of ASK1 activation in MMP-1 expression and cell motility in human chondrosarcoma is largely unknown. Furthermore, BDNF is expressed in bone and cartilage [8,10]. In this study, we explored the involvement of the intracellular ASK1 signaling pathway in BDNF-induced MMP-1 production and cell migration in human chondrosarcoma. The results showed that BDNF binds to TrkB, which activates the ASK1, JNK/p38, and Sp1 pathways, leading to up-regulated MMP-1 expression, resulting in the promotion of migration of human chondrosarcoma cells.

## 2. Results

### 2.1. BDNF Expressed Is Positively Correlated with Chondrosarcoma Cells Motility

**Figure 1:**
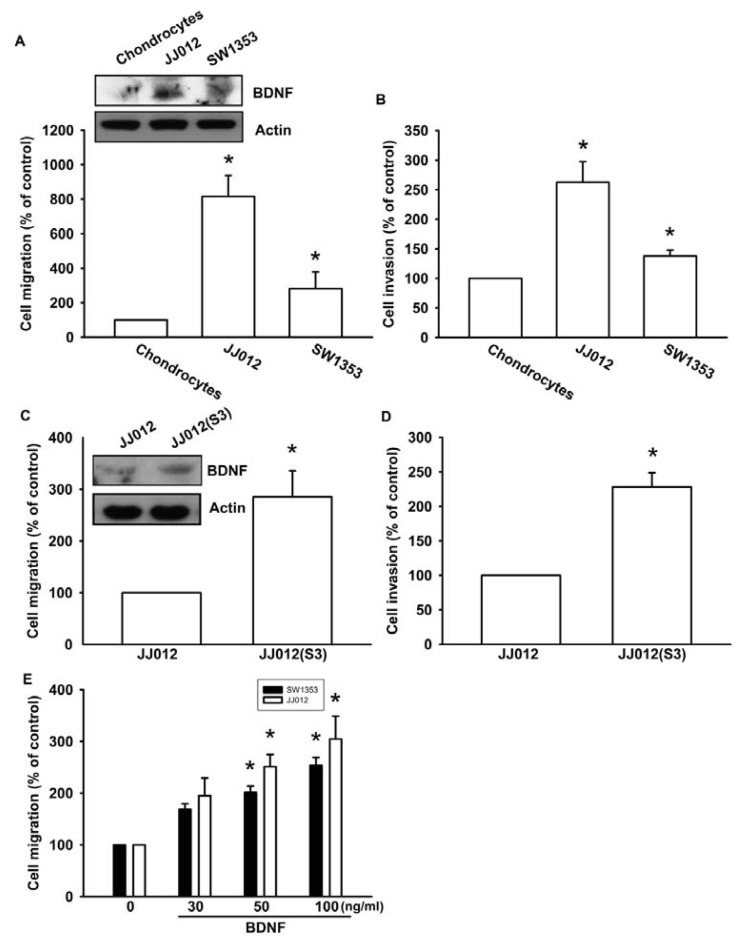
Brain-derived neurotrophic factor (BDNF) increases migration and invasion of chondrosarcoma cells. (**A** and **B**) *In vitro* cell migration, invasion, and BDNF expression of primary chondrocytes, JJ012, and SW1353 cells were measured using a Transwell assay and western blotting; (**C** and **D**) *In vitro* cell migration, invasion, and BDNF expression of JJ012 and JJ012(S3) cells were measured using the Transwell assay and western blotting; (**E**) Cells were incubated with BDNF (30–100 ng/mL), and *in vitro* migration was measured with the Transwell assay after 24 h. Results are expressed as the mean ± S.E. *p < 0.05 compared with control; ^#^*p* < 0.05 compared with the BDNF-treated group.

Previous studies have reported that BDNF is expressed in bone and cartilage tissues, and is able to increase cell migration and invasion activity in many human cancer cells [24,25]. We first compared BDNF levels between human normal chondrocytes and chondrosarcoma cell lines (JJ012 and SW1353). We found that BDNF protein expression was higher in chondrosarcoma cells (JJ012 and SW1353) than in normal chondrocytes (Figure 1A, upper panel). In addition, chondrosarcoma cells were more migratory and invasive than normal chondrocytes (Figure 1A,B). To further confirm BDNF-mediated cell migration in human chondrosarcoma cells, we selected JJ012 sublines that showed higher cell mobility (see Experimental Section). The invasion-prone subline JJ012(S3) had higher cell motility compared to the original JJ012 cell line (Figure 1C,D). Moreover, JJ012(S3) showed markedly increased protein expression of BDNF (Figure 1C). To examine whether BDNF induced chondrosarcoma migration, migration and invasion assays were performed in two chondrosarcoma cell lines (JJ012 and SW1353). Treating chondrosarcoma cell lines with BDNF (30–100 ng/mL) dramatically increased cell migration (Figure 1E). These results clearly demonstrated that the expression of BDNF is positively correlated with the motility of chondrosarcoma cells.

### 2.2. BDNF Increases MMP-1 Expression in Chondrosarcoma Cells

**Figure 2:**
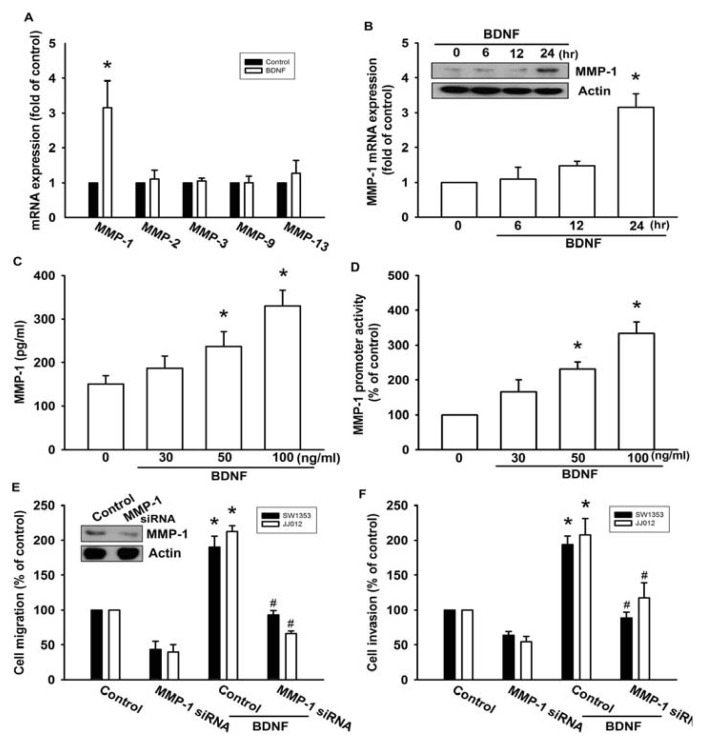
BDNF-directed migration and invasion of human chondrosarcoma cells involves up-regulation of matrix metalloproteinase-1 (MMP-1). (**A**) JJ012 cells were incubated with BDNF (50 ng/mL) for 24 h, and the mRNA levels of MMP-1, -2, -3, -9, and -13 were determined using qPCR; (**B**) JJ012 cells were incubated with BDNF (50 ng/mL) for indicated time intervals, and the protein and mRNA expressions of MMP-1 were examined by western blotting and qPCR; (**C** and **D**) JJ012 cells were incubated with various concentrations of BDNF (30–100 ng/mL) for 24 h, and MMP-1 expression was examined based on enzyme-linked immunosorbent assay (ELISA) and MMP-1 promoter activity; (**E** and **F**) Cells were transfected with MMP-1 siRNA for 24 h followed by treatment with BDNF (50 ng/mL) for 24 h, and cell migration and invasion were examined using the Transwell assay. Results are expressed as the mean ± S.E. *p < 0.05 compared with control; ^#^*p* < 0.05 compared with BDNF-treated group.

A previous study showed that MMP-1, MMP-2, MMP-3, MMP-9, and MMP-13 were expressed in high quantities in human chondrosarcoma cells [16]. Therefore, we hypothesized that any of these MMPs might be involved in BDNF-directed chondrosarcoma migration and invasion activity. Stimulation of JJ012 cells with BDNF induced the mRNA expression of MMP-1, but not the other MMPs (Figure 2A). In addition, treatment of cells with BDNF increased mRNA and protein expressions of MMP-1 in a time-dependent manner (Figure 2B). We further confirmed that treatment of JJ012 cells with BDNF (30–100 ng/mL) for 24 h induced MMP-1 production in culture medium in a concentration-dependent manner (Figure 2C). After transfection with the MMP-1 promoter luciferase vector for 24 h, the luciferase activity was increased as a result of treatment with BDNF in a concentration-dependent manner (Figure 2D). Finally, we investigated whether the loss of MMP-1 might affect BDNF-induced cell migration. Transfection of cells with MMP-1 small interfering RNA (siRNA) markedly inhibited basal migration and invasion as well as MMP-1 expression (Figure 2E,F). In addition, MMP-1 siRNA reduced BDNF-induced cell migration and invasion activity (Figure 2E,F). These data suggest that BDNF-induced migration and invasion activity may occur via activation of MMP-1 expression.

### 2.3. The TrkB Receptor Is Involved in BDNF-Mediated MMP-1 Up-Regulation and Cell Migration of Chondrosarcoma Cells

**Figure 3:**
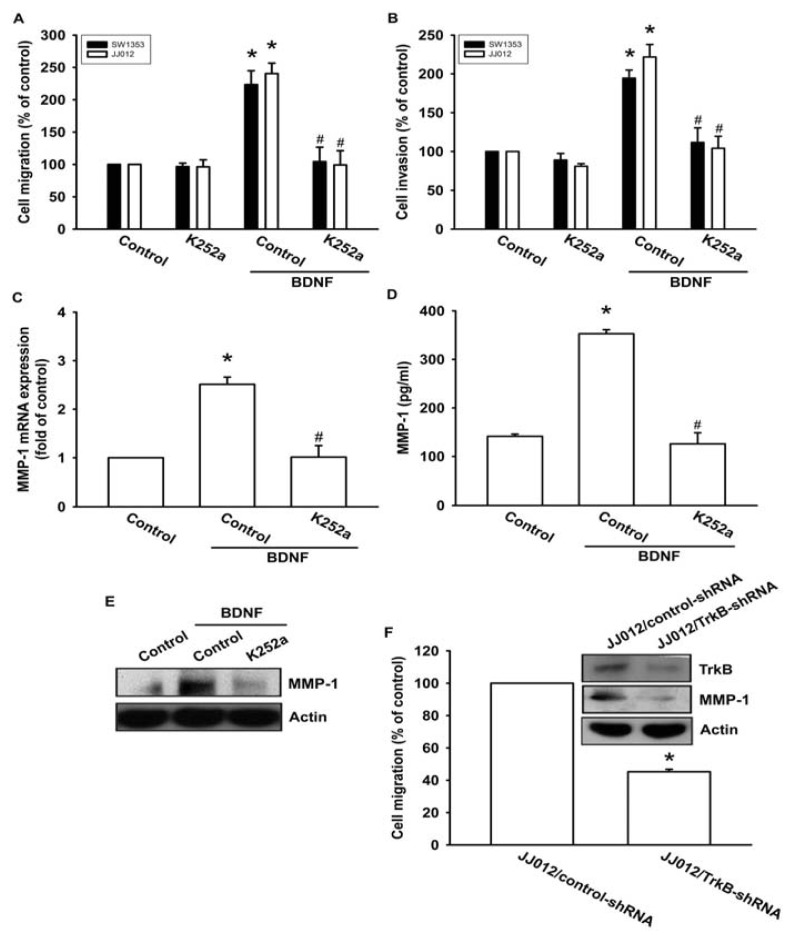
BDNF increases cell migration and MMP-1 expression through the TrkB receptor. (**A**–**E**) Cells were pretreated with K252a (50 nM) for 30 min followed by stimulation with BDNF (50 ng/mL), and *in vitro* migration, invasion, and MMP-1 expression levels were measured using Transwell, qPCR, ELISA, and western blotting; (**F**) The protein levels of TrkB and MMP-1, and the *in vitro* migration activity of JJ012/control-shRNA and JJ012/TrkB-shRNA cells were measured by western blotting and Transwell assays. Results are expressed as the mean ± S.E. *p < 0.05 compared with control; ^#^*p* < 0.05 compared with the BDNF-treated group.

Previous studies have shown that BDNF exerts its effects through interactions with a specific TrkB receptor [26,27]. Therefore, a Trk receptor-specific inhibitor, K252a, was used to examine whether the TrkB receptor might be involved in BDNF-mediated cell migration [6,28]. The results showed that pretreatment of cells with K252a reduced BDNF-induced increases in cell migration and invasion activity (Figure 3A,B). In addition, pretreatment of cells with K252a for 30 min followed by incubation with BDNF for 24 h resulted in reduced BDNF-induced MMP-1 production and mRNA expression (Figure 3C–E). To determine the specific role that the TrkB receptor plays in BDNF-mediated MMP-1 up-regulation during chondrosarcoma migration, we took advantage of JJ012 cells that stably expressed TrkB small hairpin RNA (JJ012/TrkB-shRNA), and an empty vector plasmid was used as a negative control (JJ012/control-shRNA). JJ012 cell migration, as well as TrkB and MMP-1 protein expressions, were significantly reduced in the TrkB knockdown (Figure 3F). These results indicate that the TrkB receptor is involved in BDNF-mediated MMP-1 up-regulation and cell motility in chondrosarcoma cells.

### 2.4. Involvement of ASK1 in BDNF-Induced Migration and MMP-1 Expression

**Figure 4:**
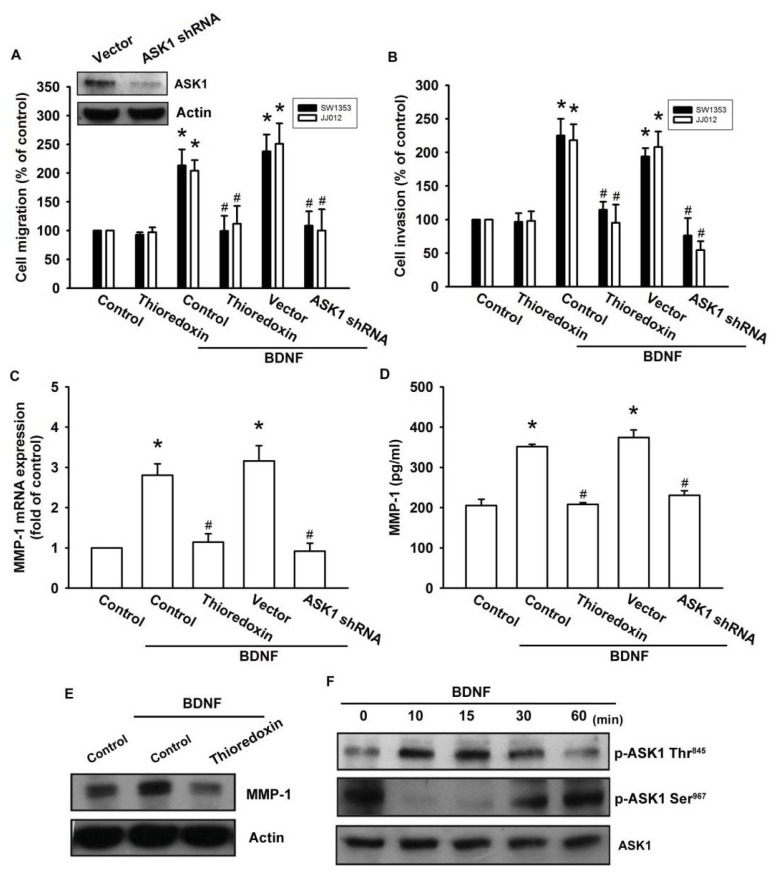

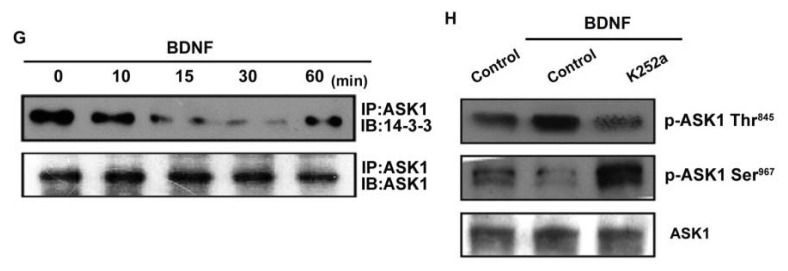
Apoptosis signal-regulating kinase 1 (ASK1) is involved in BDNF-induced migration and MMP-1 expression. (**A**–**E**) Cells were pretreated for 30 min with thioredoxin (200 ng/mL) or were transfected with ASK1 shRNA for 24 h followed by stimulation with BDNF (50 ng/mL), and *in vitro* migration and invasion or MMP-1 expression was measured by Transwell, qPCR, ELISA, and western blotting; (**F**) JJ012 cells were incubated with BDNF (50 ng/mL) for the indicated time intervals, and ASK1 phosphorylation was examined by western blotting; (**G**) JJ012 cells were incubated with BDNF (50 ng/mL) for the indicated time intervals, and were then immunoprecipitated (IP) with anti-ASK1. The IP complexes were subjected to immunoblotting (IB) with anti-14-3-3; (**H**) JJ012 cells were pretreated for 30 min with K252a for 30 min followed by stimulation with BDNF (50 ng/mL) for 10 min, and ASK1 phosphorylation was determined by western blotting. Results are expressed as the mean ± S.E. *p < 0.05 compared with control; ^#^*p* < 0.05 compared with the BDNF-treated group.

ASK1 is a member of the MKKK family, and is thus involved in the MAPK pathway [29]. ASK1 has been shown to be involved in cancer, diabetes, cardiovascular, and neurodegenerative diseases [29]. In addition, ASK1 plays a crucial role in regulating MMP-1 expression [23]. Therefore, we hypothesized that ASK1 might be involved in BDNF-directed cell migration and invasion activity in chondrosarcoma. The results showed that BDNF-induced migration and invasion ability, as well as MMP-1 up-regulation of chondrosarcoma cells, were greatly reduced by pretreatment with the ASK1 inhibitor thioredoxin [30] (Figure 4A–E). Because thioredoxin is a non-specific ASK1 inhibitor, the effects of ASK1-specific shRNA were further investigated. Transfection of cells with ASK1 shRNA reduced ASK1 protein expression (Figure 4A; upper panel). Moreover, transfection of cells with ASK1 shRNA inhibited BDNF-induced motility and MMP-1 expression in chondrosarcoma cells (Figure 4A–D). Phosphorylation at Ser^967^ was previously reported to be essential for the association between ASK1 and the 14-3-3 protein, which attenuates ASK1 activity [31]. In the present study, we found that BDNF induced transient dephosphorylation of ASK1 on Ser^967^ after 10~30 min, and recovery was observed at 60 min (Figure 4F). In addition, BDNF induced ASK1 phosphorylation on Ser^845^ at 10~30 min (Figure 4F). A previous study demonstrated that the dissociation of ASK1 from the inhibitory protein 14-3-3 could lead to ASK1 activation [31]. Therefore, we used a co-immunoprecipitation assay to determine whether BDNF-induced ASK1 dephosphorylation might be accompanied by dissociation of ASK1-14-3-3 complexes. The results showed that BDNF rapidly induced ASK1 dissociation from the 14-3-3 protein (Figure 4G). On the other hand, pretreatment of cells with K252a reduced BDNF-induced ASK1 phosphorylation of Thr^845^ and dephosphorylation of Ser^967^ (Figure 4H). These results suggest that BDNF acts through TrkB and the ASK1-dependent signaling pathway to enhance cell migration and MMP-1 production in human chondrosarcoma cells.

### 2.5. The JNK and p38 Signaling Pathways Are Involved in BDNF-Mediated MMP-1 Up-Regulation and Cell Motility of Chondrosarcoma Cells

**Figure 5:**
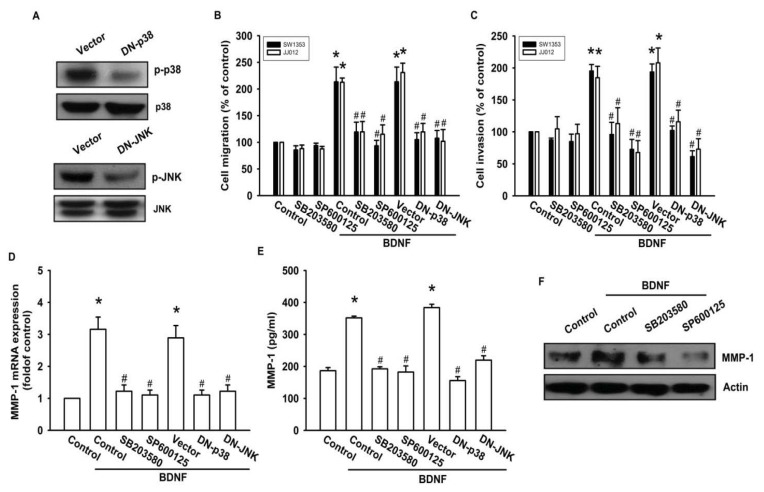

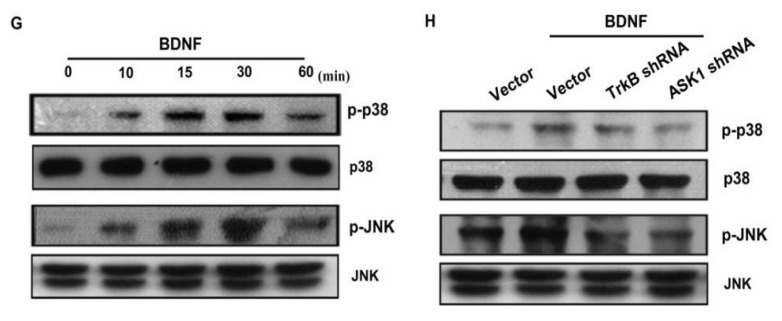
BDNF increases cell motility and MMP-1 expression through the p38 and c-jun N-terminal kinase (JNK) pathways. (**A**) JJ012 cells were transfected with dominant negative (DN) mutants of p38 and JNK for 24 h, and p38 and JNK phosphorylation were examined by western blotting; (**B**–**F**) Cells were pretreated for 30 min with SB203580 (10 μM) and SP600125 (10 μM) or were transfected with DN mutants of p38 and JNK for 24 h followed by stimulation with BDNF (50 ng/mL), and *in vitro* migration and invasion or MMP-1 expression were measured by Transwell, qPCR, ELISA, and western blotting; (**G**) JJ012 cells were incubated with BDNF (50 ng/mL) for the indicated time intervals, and p38 and JNK phosphorylation were examined by western blotting; (**H**) JJ012 cells were transfected with TrkB or ASK1 shRNA for 24 h followed by stimulation with BDNF (50 ng/mL) for 30 min, and p38 and JNK phosphorylation were determined by western blotting. Results are expressed as the mean ± S.E. *p < 0.05 compared with control; ^#^*p* < 0.05 compared with the BDNF-treated group.

As a member of the MKKK family, ASK1 activates the JNK and p38 pathways via MAPK kinase (MKK)4/7 and MKK3/6, respectively [18,32]. We examined the role of JNK and p38 in mediating BDNF-induced cell migration and invasion activity using the specific JNK inhibitor SP600125, and the specific p38 inhibitor SB203580. As shown in Figure 5B–F, pretreatment of cells for 30 min with SP600125 and SB203580 or transfection of cells for 24 h with JNK or p38 dominant negative mutants reduced BDNF-induced increases in cell motility and MMP-1 expression. We also examined the role of JNK and p38 dominant negative mutants on phosphorylation of JNK and p38. Transfection of JNK or p38 dominant negative mutants reduced JNK or p38 phosphorylation, respectively (Figure 5A). To directly confirm the crucial role that JNK and p38 play in BDNF-mediated cell migration, we measured the level of JNK and p38 phosphorylation in response to BDNF. Stimulation of JJ012 cells with BDNF resulted in time-dependent phosphorylation of JNK and p38 (Figure 5G). We next evaluated the relationship among TrkB, ASK1, and JNK/p38 in the BDNF-mediated signaling pathway, and found that transfection of cells with TrkB or ASK1 shRNA markedly inhibited BDNF-induced JNK and p38 phosphorylation (Figure 5H). Based on these results, BDNF appears to act via the TrkB receptor and the ASK1 and JNK/p38-dependent signaling pathways to enhance cell migration and MMP-1 production in human chondrosarcoma cells.

### 2.6. Stimulating Protein 1 (Sp1) Is Involved in BDNF-Induced Cell Migration and MMP-1 Expression

**Figure 6:**
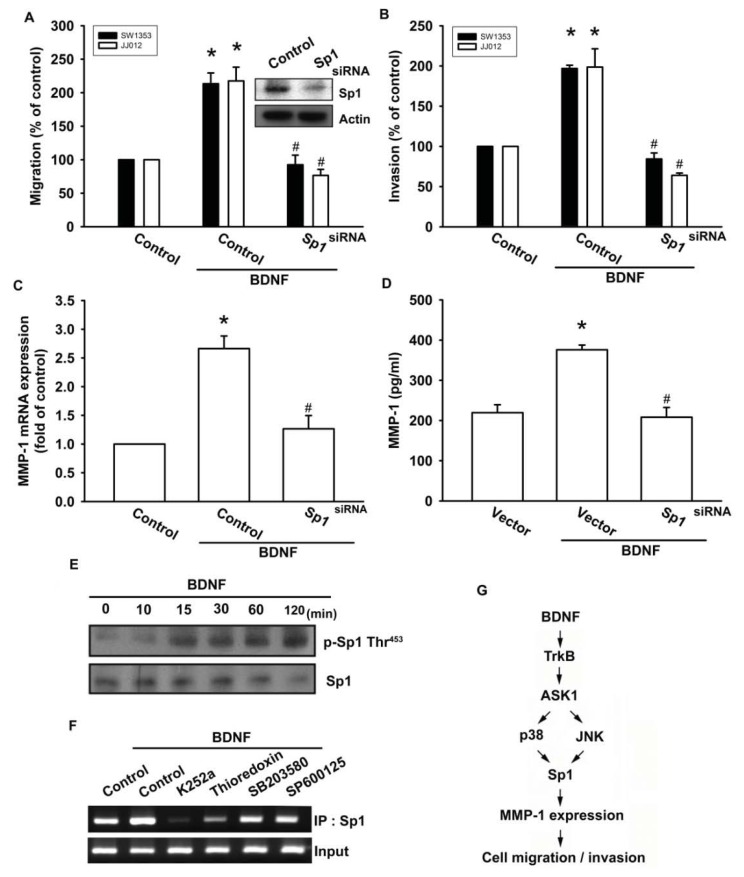
BDNF induces cell motility and MMP-1 up-regulation through Sp1. (**A**–**D**) Cells were transfected with Sp1 siRNA for 24 h followed by treatment with BDNF (50 ng/mL) for 24 h, and *in vitro* migration, invasion and MMP-1 expression were measured by Transwell, qPCR, and ELISA; (**E**) JJ012 cells were incubated with BDNF (50 ng/mL) for the indicated time intervals, and Sp1 phosphorylation was examined by western blotting; (**F**) JJ012 cells were pretreated with K252a, thioredoxin, SB203580, or SP600125 for 30 min, stimulated with BDNF (50 ng/mL) for 60 min, and the chromatin immunoprecipitation assay was then performed. Chromatin was immunoprecipitated with anti-Sp1. One percent of the precipitated chromatin was assayed to verify equal loading (input). Results are expressed as the mean ± S.E. *p < 0.05 compared with control; ^#^*p* < 0.05 compared with the BDNF-treated group; (**G**) Schematic diagram of the signaling pathways involved in BDNF-mediated enhanced cell migration and invasion activity in human chondrosarcoma cells. BDNF increases MMP-1 expression by binding to the TrkB receptor and activating ASK1 and JNK/p38, which enhances binding of Sp1 to the Sp1 site. This results in the transactivation of MMP-1 expression.

Sp1 is the first identified transcription factor that activates a broad spectrum of cellular and viral genes. It belongs to the Sp/KLF-like factor family of transcription factors that bind to the GC-rich promoter element through three Cys2His2-type zinc-fingers. It is ubiquitously expressed in both normal and cancerous tissues and has multiple functions. Multiple human cancer types have been found to be associated with the expression of Sp1 and MMPs, including glioma cells [33], lung cancer cells [34], and ovarian cancer [35]. Therefore, we next examined whether Sp1 activation might be involved in the signal transduction pathway leading to migration, invasion, and MMP-1 expression caused by BDNF. Transfection of cells with Sp1 siRNA reduced Sp1 expression (Figure 6A upper panel). In addition, transfection of chondrosarcoma cells for 24 h with Sp1 siRNA markedly inhibited BDNF-induced cell migration, invasion, and MMP-1 expression (Figure 6A–D). Phosphorylation of Sp1 at Thr^453^ was previously shown to enhance binding of Sp1 with its target sequence [36]. Therefore, we employed an antibody against phosphorylated Sp1 Thr^453^ to examine Sp1 phosphorylation. Treatment of JJ012 cells with BDNF at various time intervals resulted in Sp1 Thr^453^ phosphorylation (Figure 6E). We further investigated whether Sp1 binds to the Sp1 element on the MMP-1 promoter after BDNF stimulation. The *in vivo* recruitment of Sp1 to the MMP-1 promoter (−2339 to −2096) was assessed using a chromatin immunoprecipitation assay [37]. *In vivo* binding of Sp1 to the Sp1 element of the MMP-1 promoter occurred after BDNF stimulation (Figure 6F). BDNF induction of Sp1 binding to the MMP-1 promoter was attenuated by K252a, thioredoxin, SB203580, and SP600125 (Figure 6F). Therefore, activation of the TrkB receptor and the ASK1, JNK/p38, and Sp1 pathways are required for BDNF-induced cell migration and MMP-1 production in human chondrosarcoma cells.

## 3. Discussion

Chondrosarcoma are rare but deadly forms of bone cancer that account for nearly 26% of all bone cancers [38]. Unlike other mesenchymal malignancies, such as osteosarcoma and Ewing’s sarcoma that show dramatic increases in long-term survival in response to systemic chemotherapy, chondrosarcoma continues to have a poor prognosis [39]. Therefore, it is important to develop effective adjuvant therapy for preventing chondrosarcoma metastasis. However, the effect of BDNF in human chondrosarcoma migration remains largely unknown. Several lines of evidence in this study demonstrated that the expression of BDNF was associated with a metastatic phenotype of chondrosarcoma cells. First, the expression of BDNF was significantly higher in chondrosarcoma cells than in normal chondrocytes, and correlated strongly with cell motility. Second, treatment with exogenous BDNF increased the migration of chondrosarcoma cells. Third, over-expression of TrkB shRNA inhibited the migratory ability of chondrosarcoma cells. These data suggest that BDNF is a novel marker for the metastasis of human chondrosarcoma.

Tumor metastasis involves many steps in malignant cancers depending on the capacity to invade, metastasize, and promote the angiogenic host response. Metastasis is generally associated with a poor clinical outcome. In addition, the most critical characteristic of malignant tumor progression is the ability of metastatic cancer cells to dissolve basement membranes and the ECM. This degradative process is largely mediated by MMPs [40]. MMP-1, -2, -3, -9, and -13 were shown to correlate with malignant grade and metastasis [41,42]. Of all MMPs, MMP-1 is the dominant metalloproteinase involved in the motility of human chondrosarcoma [43]. Indeed, several lines of evidence in the current study confirmed that MMP-1 is involved in BDNF-induced cell migration of human chondrosarcoma cells. First, BDNF induced MMP-1 expression and secretion in human chondrosarcoma cells without significantly changing the expression of MMP-2, -3, -9, and -13 mRNAs. Second, BDNF-induced cell migration and invasion were inhibited by MMP-1-specific siRNA. Therefore, MMP-1 might be the BDNF-responsive mediator, which causes the degradation of the ECM, resulting in subsequent cancer migration and invasion activity. Previous studies have also shown that MMP-1 is an important mediator in cancer metastasis. In prostate and breast cancer, RANKL induced cell motility through MMP-1 up-regulation [44], whereas thromboxane A-enhanced lung cancer migration was shown to involve MMP-1 expression [45]. Furthermore, G-protein coupled receptor 64 also promoted MMP-1 expression and cell motility of Ewing sarcomas [46]. Collectively, these results suggest that MMP-1 is an important target for treating cancer metastasis.

ASK1 is a member of the MKKK family, and as such, forms part of the MAPK pathway. ASK1 activity is regulated by multiple mechanisms, including phosphorylation and interactions with various proteins, and it is also an upstream molecule of JNK and p38, which have been shown to be involved in the regulation of gene expressions [47]. In the present study, we found that pretreatment of cells with ASK1, JNK, or p38 inhibitors antagonized BDNF-induced cell migration and MMP-1 expression. This effect was further confirmed by the fact that ASK1 shRNA or JNK and p38 mutants inhibited BDNF-enhanced cell motility and MMP-1 expression. Therefore, our results provide evidence that BDNF up-regulates MMP-1 expression and cell migration in human chondrosarcoma cells via the ASK1 and JNK/p38 signaling pathways. In addition to cancer metastasis, a similar signal pathway involving ASK1-dependent JNK/p38 activation was reported to be involved in IL-6 induced angiogenesis and metastasis in osteosarcomas [48]. Importantly, the present study provides the first evidence that ASK1-dependent JNK/p38 is a downstream molecule of the BDNF signaling pathway. However, whether this pathway is common in BDNF-mediated gene expression needs further investigation.

## 4. Experimental Section

### 4.1. Materials

Protein A/G beads, anti-mouse and anti-rabbit IgG-conjugated horseradish peroxidase, rabbit polyclonal antibodies specific for BDNF, TrkB, ASK1, p-p38, p38, p-JNK, JNK, Sp1, MMP-1, and β-actin, siRNAs against Sp1, MMP-1, ASK1 shRNA, and control shRNA plasmids were purchased from Santa Cruz Biotechnology (Santa Cruz, CA, USA). We found that transfection of cells with MMP-1, TrkB, or Sp1 siRNA reduced MMP-1, TrkB, and Sp1 expression, respectively, both in the absence and presence of BDNF (Figures 2E,3F,6A, and Supplemental Figure S1). Rabbit polyclonal antibodies specific to ASK1 phosphorylated at Thr^845^ and Ser^967^ were purchased from Cell Signaling and Neuroscience (Danvers, MA, USA). The rabbit polyclonal antibody specific to SP1 phosphorylated at Thr^453^ was purchased from Abcam (Cambridge, MA, USA). The recombinant human BDNF was purchased from R&D Systems (Minneapolis, MN, USA). The p38 dominant negative mutant was provided by Dr. J. Han (University of Texas Southwestern Medical Center, Dallas, TX, USA). The JNK dominant negative mutant was provided by Dr. M. Karin (University of California, San Diego, CA, USA). The pGL2 luciferase reporter vector and the luciferase assay kit were purchased from Promega (Madison, WI, USA). All other chemicals were purchased from Sigma-Aldrich (St. Louis, MO, USA).

### 4.2. Cell Culture

The human chondrosarcoma cell line (JJ012) was kindly provided from the laboratory of Dr. Sean P. Scully (University of Miami School of Medicine, Miami, FL, USA) [49]. The TrkB shRNA-expressing cells were puromycin selected. Surviving cells were removed and expanded to establish clonal cell populations. For monolayer growth curves, 10^4^ cells were plated in 6-well plates and grown for one to six days. Cells were trypsinized and cell numbers were counted [50]. Cells were cultured in Dulbecco’s modified Eagle’s medium (DMEM)/α-MEM supplemented with 10% fetal bovine serum (FBS) and maintained at 37 °C in a humidified atmosphere of 5% CO_2_. The human chondrosarcoma cell line (SW1353) was purchased from the American Type Culture Collection. The cells were cultured in DMEM supplemented with 10% FBS. These cells were maintained at 37 °C in a humidified atmosphere of 5% CO_2_.

Primary cultures of human chondrocytes were isolated from articular cartilage as previously described [51]. The cells were grown in plastic cell culture dishes in 95% air/5% CO_2_ in DMEM supplied with 20 mM HEPES, 10% heat-inactivated FBS, 2 mM-glutamine, 100 U/mL penicillin, and 100 μg/mL streptomycin.

### 4.3. Migration and Invasion Assay

The migration assay was performed using Transwell (Costar, NY, USA; 8-μm pore size) in 24-well dishes. For the invasion assay, filters were precoated with 30 μL Matrigel basement membrane matrix (BD Biosciences, Bedford, MA, USA) for 30 min. The same following procedures were used for both the migration and invasion assays. Before the migration assay was performed, cells were pretreated for 30 min with different concentrations of inhibitors, including K252a, thioredoxin, SP600125, SB203580, or vehicle control (0.1% dimethyl sulfoxide). Approximately 1 × 10^4^ cells in 200 μL of serum-free medium were placed in the upper chamber, and 300 μL of the serum-free medium containing BDNF was placed in the lower chamber. The plates were incubated for 24 h at 37 °C in 5% CO_2_, and then cells were fixed in 3.7% formaldehyde solution for 15 min and stained with 0.05% crystal violet in phosphate-buffered saline (PBS) for 15 min. Cells on the upper side of the filters were removed with cotton-tipped swabs, and the filters were washed with PBS. Cells on the underside of the filters were examined and counted under a microscope. Each clone was plated in triplicate in each experiment, and each experiment was repeated at least three times. The number of migrating cells in each experiment was adjusted by the cell viability assay to correct for proliferation effects of the BDNF treatment (corrected migrating cell number: counted migrating cell number/percentage of viable cells) [52].

### 4.4. Establishment of Invasion-Prone Sublines

Subpopulations of JJ012 cells were selected according to their differential invasion ability, which was determined using the cell culture insert system described previously. After 24 h invasion, cells that penetrated through pores and invaded to the underside of the filters were trypsinized and harvested for a second round of selection. The original cells that did not pass through membrane pores were designated as JJ012-S0. After three rounds of selection, the invasion-prone subline was designated as JJ012-S3.

### 4.5. Western Blot Analysis

The cellular lysates were prepared as described previously [53,54]. Proteins were resolved by sodium dodecyl-polyacrylamide gel electrophoresis and transferred to Immobilon polyvinyl difluoride membranes. The blots were blocked with 4% nonfat milk for 1 h at room temperature, and were then probed with rabbit anti-human antibodies against ASK1, p-ASK1, JNK, p38, BDNF, and TrkB (1:1000) for 1 h at room temperature. After three washes, the blots were subsequently incubated with a goat anti-rabbit or goat anti-mouse peroxidase-conjugated secondary antibody (1:1000) for 1 h at room temperature. The blots were visualized by enhanced chemiluminescence using Kodak X-OMAT LS film (Eastman Kodak, Rochester, NY, USA).

### 4.6. Real-Time Quantitative Polymerase Chain Reaction (qPCR)

Total RNA was extracted from chondrosarcoma cells using a TRIzol kit (MDBio Inc., Taipei, Taiwan). The reverse transcription reaction was performed using 1 μg of total RNA that was reverse transcribed into cDNA using oligo(dT) primer [55,56]. The qPCR analysis was carried out using Taqman^®^ one-step PCR Master Mix (Applied Biosystems, Foster City, CA, USA). Two microliters cDNA templates were added to each 25 μL reaction with sequence-specific primers and Taqman^®^ probes. Sequences for all target gene primers and probes were purchased commercially, and glyceraldehyde 3-phosphate dehydrogenase (GAPDH) was used as the endogenous control to normalize expression data (Applied Biosystems). qPCR assays were carried out in triplicate on a StepOnePlus sequence detection system. The cycling conditions were 10 min polymerase activation at 95 °C followed by 40 cycles at 95 °C for 15 s and 60 °C for 60 s. The threshold was set above the non-template control background and within the linear phase of target gene amplification in order to calculate the cycle number at which the transcript was detected (denoted Ct). MMP-1 mRNA levels were normalized to GAPDH mRNA levels and expressed relative to the control using the ΔΔCt method.

### 4.7. Measurement of MMP-1 Production

Human chondrosarcoma cells were cultured in 24-well culture plates. After reaching confluence, cells were treated with MMP-1 and then incubated in a humidified incubator at 37 °C for 24 h. To examine the downstream signaling pathways involved in BDNF treatment, cells were pretreated with various inhibitors for 30 min before BDNF (50 ng/mL) administration. After incubation, the medium was removed and stored at −80 °C until the assay was performed. MMP-1 in the medium was assayed using MMP-1 enzyme immunoassay kits (R & D) according to the procedure described by the manufacturer.

### 4.8. MMP-1 Promoter Constructs

Promoter construct DNA fragments, which covered positions −1599 to −2339 bp, were amplified by PCR with chemically synthesized oligonucleotides that corresponded to nucleotides −2339 to −2319 bp of the sense strand (5′-GAGAGCTCTGGACTCAGATGC-3′) and −1621 to −1599 bp of the antisense strand (5′-CTGCGTCAAGACTGATATCTTAC-3′), relative to the transcription start site of human MMP-1 genomic DNA [57]. DNA fragments were purified using the HiYield Gel/PCR Extraction Kit (RBC Bioscience, Taipei, Taiwan) according to manufacturer recommendations, and were cloned into the pcDNA3.1/V5-His-TOPO vector (Invitrogen, Carlsbad, CA, USA). The PCR products were digested with *Xho*I and *Kpn*I. To create MMP-1-luc, the same sequence was inserted into the *Xho*I- and *Kpn*I-digested pGL3-basic vector (Promega). For the MMP-1 promoter assay, JJ012 cells were transfected with the MMP-1-luc reporter plasmid using Lipofectamine 2000 according to manufacturer recommendations. Twenty-four hours after transfection, the cells were treated with inhibitors for 30 min, and then BDNF or vehicle was added for a further 24 h. Cell extracts were then prepared, and luciferase and β-galactosidase activities were measured.

### 4.9. Chromatin Immunoprecipitation Assay

Chromatin immunoprecipitation analysis was performed as described previously [58]. DNA that was immunoprecipitated with anti-Sp1 antibody was purified and extracted with phenol-chloroform. The purified DNA pellet was subjected to PCR. PCR products were then resolved by 1.5% agarose gel electrophoresis and visualized with UV light. The primers 5′-GAGAGCTCTGGACTCAGATGC-3′ and 5′-CTGTCCCATCTGGCAAGAGCC-3′ were utilized to amplify across the MMP-1 promoter region (nucleotides −2339 to −2096 bp).

### 4.10. Statistical Analysis

Data are presented as mean ± standard error of the mean (SEM). Statistical analyses between two samples were performed using the Student’s *t*-test. Statistical comparisons of more than two groups were performed using one-way analysis of variance with Bonferroni’s post-hoc test. In all cases, *p* < 0.05 was considered significant.

## 5. Conclusions

The prognosis of patients with chondrosarcoma distant metastasis is generally considered very poor; hence, preventing human chondrosarcoma metastasis is currently an important issue. The current study demonstrated that BDNF increases MMP-1 expression by binding to the TrkB receptor and activating the ASK1, JNK/p38, and Sp1-dependent pathways, thereby enhancing the migration and invasion activity of human chondrosarcoma cells (Figure 6G). Furthermore, the discovery of the BDNF-mediated signaling pathway helps to increase understanding of the mechanism underlying human chondrosarcoma metastasis, which could lead to development of effective therapy in the future.

## Supplementary Information
