# Supplementary material for: Apoptosis Signal-Regulating Kinase 1 Is Involved in Brain-Derived Neurotrophic Factor (BDNF)-Enhanced Cell Motility and Matrix Metalloproteinase 1 Expression in Human Chondrosarcoma Cells
Source: Int J Mol Sci. 2013 Jul 25;14(8):15459–78. doi: 10.3390/ijms140815459 (PMC3759868; doi:10.3390/ijms140815459)
Supplement: Supplementary file 1 [file ijms-14-15459-s001.pdf]

Supplementary Information

Figure S1. MMP-1 siRNA, TrkB shRNA or Sp1 siRNA reduced MMP-1, TrkB, and Sp1 expression. Cells were transfected with MMP-1 siRNA, TrkB shRNA, or Sp1 siRNA for 24 h followed by stimulation with BDNF for 24 h. The MMP-1, TrkB, or Sp1 protein expression was measured by western blotting.

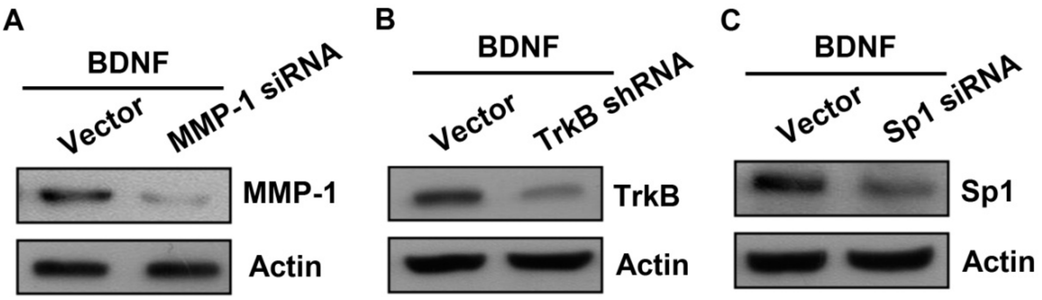

© 2013 by the authors; licensee MDPI, Basel, Switzerland. This article is an open access article distributed under the terms and conditions of the Creative Commons Attribution license (http://creativecommons.org/licenses/by/3.0/).
